# Effect of γ-Cyclodextrin Inclusion Complex on the Absorption of R-α-Lipoic Acid in Rats

**DOI:** 10.3390/ijms160510105

**Published:** 2015-05-04

**Authors:** Ryota Uchida, Kosuke Iwamoto, Suetada Nagayama, Atsushi Miyajima, Hinako Okamoto, Naoko Ikuta, Hiroshi Fukumi, Keiji Terao, Takashi Hirota

**Affiliations:** 1 Department of Biopharmaceutics, Faculty of Pharmaceutical Science, Tokyo University of Science, 2641 Yamazaki, Noda-shi, Chiba 278-8510, Japan; E-Mails: j3b13702@ed.tus.ac.jp (R.U.); gan.ln284@gmail.com (K.I.); j3a08085@ed.noda.tus.ac.jp (S.N.); miyajima@my-pharm.ac.jp (A.M.); 2 CycloChem Bio Co., Ltd., KIBC654R 5-5-2 Minatojima-minamimachi Chuo-ku, Kobe 650-0047, Japan; E-Mails: hinako.okamoto@cyclochem.com (H.O.); fukumihiroshi@aol.com (H.F.); keiji.terao@cyclochem.com (K.T.); 3 Graduate School of Medicine, Kobe University, 7-5-2 Kusunoki-cho Chuo-ku, Kobe 650-0017, Japan; E-Mail: naoko.ikuta@people.kobe-u.ac.jp

**Keywords:** γ-cyclodextrin, inclusion complex, R-α-lipoic acid, pharmacokinetic-profile, absorption, intraduodenal administration, rats, X-ray imaging

## Abstract

R-α-lipoic acid (RLA) is an endogenous organic acid, and works as a cofactor for mitochondrial enzymes and as a kind of antioxidant. Inclusion complexes of RLA with α-, β- or γ-cyclodextrins (CD) were prepared and orally administered as a suspension to rats. Among them, RLA/γ-CD showed the highest plasma exposure, and its area under the plasma concentration-time curve (AUC) of RLA was 2.2 times higher than that after oral administration of non-inclusion RLA. On the other hand, the AUC after oral administration of non-inclusion RLA and RLA/γ-CD to pylorus-ligated rats did not differ. However, the AUC after intraduodenal administration of RLA/γ-CD was 5.1 times higher than that of non-inclusion RLA, and was almost comparable to the AUC after intraduodenal administration of RLA-Na solution. Furthermore, the AUC after intraduodenal administration of RLA/γ-CD was not affected by biliary ligation or co-administration of an amylase inhibitor. These findings demonstrated that RLA was absorbed from the small intestine effectively when orally administered as a γ-CD inclusion complex, which could be easily dissolved in the lumen of the intestine. In conclusion, γ-CD inclusion complex is an appropriate formulation for supplying RLA as a drug or nutritional supplement with respect to absorption.

## 1. Introduction

α-Lipoic acid (LA; 5-(1,2-dithiolan-3-yl) pentanoic acid) is a sulfur-containing organic acid derived from octanoic acid [1,2]. LA is unique among natural antioxidants in its ability to fulfil all of these requirements, making it a potentially highly effective therapeutic agent for a number of conditions in which oxidative damage has been implicated [3]. LA’s antioxidant properties consist of the following: (1) Its capacity to scavenge reactive oxygen species (ROS) directly; (2) Its ability to regenerate endogenous antioxidants, such as glutathione and vitamins E and C; and (3) Its metal-chelating activity, resulting in reduced ROS production. Moreover, LA plays a pivotal role as antioxidant and metabolic component of some enzymatic complexes involved in glucose metabolism of different cell types [4]. LA has two sulfur atoms, one each at the C6 and C8 carbons, connected by a disulfide bond, and because the C6 carbon is chiral, LA exists as two enantiomers (R and S forms of LA). R-α-lipoic acid (RLA) is biosynthesized from octanoic acid in mitochondria [5,6], and works as a cofactor of various mitochondrial respiratory chain enzymes such as pyruvate, α-ketoglutarate and branched-chain α-ketoacid dehydrogenases [7]. Under normal physiological conditions, it is supplied by biosynthesis and food ingestion. On the other hand, S-α-lipoic acid (SLA) is not a naturally occurring compound [8]. Both forms seem to have different potencies. The *R*-form is more potent than the *S*-form in its ability to stimulate glucose uptake in L6 myotubes, as well as to increase insulin-stimulated glucose uptake in obese Zucker rats [9].

Several clinical trials showed that LA was effective for the treatment of burning mouth syndrome [10], peripheral artery disease [11], metabolic syndrome [12], and liver diseases [13]. Furthermore, LA is thought to decrease the blood glucose level and body weight [14,15,16]. Hence, a racemic form of LA (R/S-LA) is widely sold world-wide as a nutritional supplement to retard aging or to prevent obesity, and is used in Germany as a therapeutic drug for diabetic neuropathy [17].

Cremer *et al*. calculated the 50% lethal dose and the no-observed-adverse-effect level for R/S-LA in rats to be more than 2000 mg/kg and 61.9 mg/kg/day, respectively, based on acute and subchronic toxicity studies [18]. Therefore, R/S-LA has been administered at doses between 600 and 1800 mg/day in many clinical trials [19,20,21,22]. On the other hand, Gal reported that SLA was slightly more toxic than RLA in thiamine deficient rats [23]. Thus, RLA would be preferred to R/S-LA as a drug or nutritional supplement in the light of safety. Unfortunately, however, since RLA is physicochemically unstable under conditions such as acid and heat, it is difficult to prepare an optically pure RLA formulation. In the United States, even though RLA sodium salt (RLA-Na) is sold on the market as stabilized RLA, its stability is still insufficient [24].

Cyclodextrins (CDs) are cyclic oligosaccharides consisting of d-glucopyranose. Naturally occurring CDs are classified into 3 types by the number of d-glucopyranoses, α-CD (six d-glucopyranoses), β-CD (seven) and γ-CD (eight). These have different features, such as molecular weight and solubility in water [25], and each has a hydrophobic cavity inside the molecule in which an insoluble or poorly soluble compound can be easily lodged as a guest compound to create a CD inclusion complex. In general, the CD inclusion complex can improve the guest compound’s water solubility and physicochemical stability, and in addition mask bad taste and odor. Due to these properties, CDs are widely used in drugs, foods, and cosmetics.

γ-CD was thought to be appropriate for oral formulation because γ-CD has higher water solubility, and is more easily hydrolyzed by α-amylase compared with α-CD or β-CD [25], which would decrease the risk of gastrointestinal disturbance. In fact, Lina *et al*. reported that α-CD caused subchronic oral toxicity such as persistent diarrhea, and decreasing food consumption [26]. JECFA, the Joint FAO/WHO Expert Committee on Food Additives, places a limit for acceptable daily intake of β-CD as a food additive. In addition, in 2013, Ikuta *et al*. prepared various RLA/CD, and examined the properties of the RLA/CDs, and determined which was superior in pharmaceutical preparations such as stability to acid and heat, and the yield of complexation [24]. Based on such parameters, RLA/γ-CD was concluded to be a preferable formulation compared to RLA/α-CD, RLA/β-CD, RLA-Na and RLA. However, whether the γ-CD inclusion complex influences the pharmacokinetic profile of RLA remains unknown.

We considered that effective absorption as RLA would be very important in the light of efficacy and safety. Thus, in the present study, we compared the exposures after oral administration of α-, β-, γ-CD inclusion complexes of RLA and non-inclusion RLA to rats, in order to find the most appropriate CD. In addition, we examined where and why RLA in the γ-CD inclusion complex was absorbed more effectively than RLA alone.

## 2. Results

### 2.1. Absorption after Oral Administration of RLA and Its CD Inclusion Complexes

**Figure 1:**
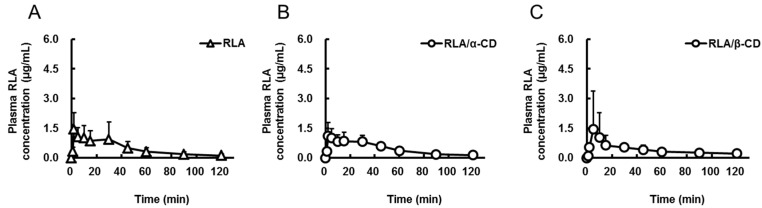

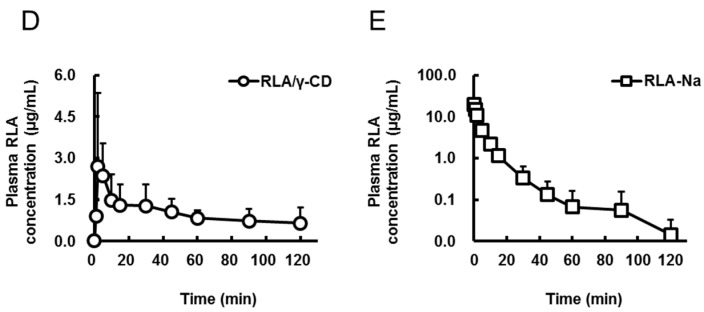
Plasma concentration-time profiles of R-α-lipoic acid after oral administration of R-α-lipoic acid (**A**); R-α-lipoic acid/α-cyclodextrin inclusion complex (**B**); R-α-lipoic acid/β-cyclodextrin inclusion complex (**C**) and R-α-lipoic acid/γ-cyclodextrin inclusion complex (**D**) and after intravenous administration of R-α-lipoic acid sodium salt (**E**) to rats. Data are shown as mean ± standard deviation (*n* = 6).

The plasma concentrations of RLA were measured after oral administration of non-inclusion RLA or RLA/CDs (20 mg RLA/kg, 2 mL/kg, Figure 1A–D) or intravenous administration of RLA-Na (5 mg RLA/kg, 1 mL/kg, Figure 1E) to rats, and the pharmacokinetic parameters were calculated (Table 1). Although there were no significant differences in the *C*_max_ and *T*_max_ among the groups after oral administration, the *AUC*_0–t_ (area under the plasma concentration *vs.* time curve) of RLA after administration of RLA/γ-CD was higher than after administration of the other compounds (*p* < 0.05, Table 1). The value of *AUC*_po_/*AUC*_iv_ until 120 min for the RLA/γ-CD group was more than twice higher than those for the others.

**Table 1:** Pharmacokinetic parameters of R-α-lipoic acid after oral administration of R-α-lipoic acid or R-α-lipoic acid/cyclodextrin inclusion complexes and intravenous administration of R-α-lipoic acid sodium salt to rats.

Formulation	RLA	RLA/α-CD	RLA/β-CD	RLA/γ-CD	RLA-Na
Route	po	po	po	po	iv
Dose as amount of RLA (mg/kg)	20	20	20	20	5
*C*_max_ or *C*_0_ (µg/mL)	1.7 ± 0.9	1.4 ± 0.6	1.6 ± 1.9	3.4 ± 2.5	19.5 ± 3.3
*T*_max_ (min)	11.8 ± 14.1	10.7 ± 10.7	33.3 ± 44.0	9.0 ± 10.7	not determined
*AUC*_0-t_ (µg·min/mL)	56 ± 35 *	56 ± 12 *	50 ± 19 *	121 ± 24	96 ± 19
*AUC*_po_/*AUC*_iv_ (%)	14.6	14.6	13	31.5	not calculated

Pharmacokinetic parameters are shown as mean ± standard deviation except *AUC*_po_/*AUC*_iv_ row (*n* = 6). RLA, R-α-lipoic acid; RLA/α-CD, R-α-lipoic acid/α-cyclodextrin inclusion complex; RLA/β-CD, R-α-lipoic acid/β-cyclodextrin inclusion complex; RLA/γ-CD, R-α-lipoic acid/γ-cyclodextrin inclusion complex; RLA-Na, R-α-lipoic acid sodium salt; *C*_max_, maximum plasma concentration; *C*_0_, initial concentration; *T*_max_, time of maximum drug concentration; *AUC*_0–t_, area under the plasma concentration *vs.* time curve (from initial to last points); po, per os; iv, intravenous. *, Probability (*p*) < 0.05 compared with RLA/γ-CD. Statistical analysis was performed among the po groups by using analysis of variance by followed Tukey’s multiple comparison tests.

### 2.2. Absorption Site of RLA after Administration as RLA/γ-CD

**Figure 2:**
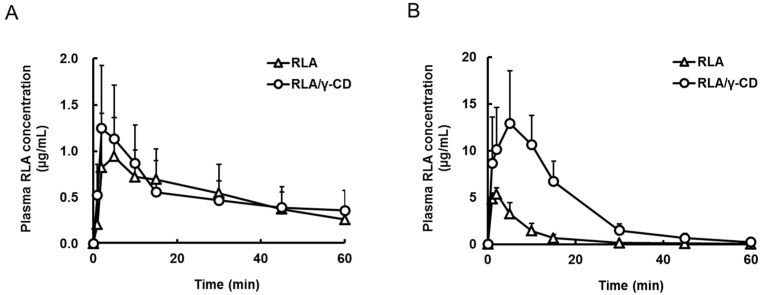
Plasma concentration-time profiles of R-α-lipoic acid after oral administration (**A**) or intraduodenal administration (**B**) of R-α-lipoic acid or R-α-lipoic acid/γ-cyclodextrin inclusion complex to pylorus ligated rats. Data are shown as mean ± standard deviation (*n* = 6).

Plasma concentrations of RLA were measured after oral or intraduodenal administration of non-inclusion RLA or RLA/γ-CD (20 mg RLA/kg, 2 mL/kg) to rats with pylorus ligation. The pharmacokinetic profiles after oral administration were understood as the absorption from the stomach, because the stomach was completely separated from the intestine. In addition, the pharmacokinetic profiles after introduodenal administration were understood as the absorption from the small intestine, because the small intestine had the largest surface area in the gastrointestinal tract. After oral administration, the *C*_max_, *T*_max_, and *AUC*_0–t_ were not significantly different between the non-inclusion RLA and RLA/γ-CD groups (Figure 2A and Table 2). On the other hand, the plasma concentrations after intraduodenal administration of RLA/γ-CD were higher than those after non-inclusion RLA, and the *C*_max_ and *AUC*_0–__t_ for RLA/γ-CD was 2.7 and 5.1 times higher than that for non-inclusion RLA, respectively (*p* < 0.05, Figure 2B, Table 2). Furthermore, the *AUC*_0-__t_ after intraduodenal administration of RLA/γ-CD was 7.1 times higher than that after oral administration (*p* < 0.05, Table 2), but the *AUC*_0-__t_ values after intraduodenal and oral administration of non-inclusion RLA were almost identical.

**Table 2:** Pharmacokinetic parameters of R-α-lipoic acid after oral administration or intraduodenal administration of R-α-lipoic acid or R-α-lipoic acid/γ-cyclodextrin inclusion complex to pylorus ligated rats.

Formulation	RLA	RLA/γ-CD	RLA	RLA/γ-CD
Route	po under PL	po under PL	id	id
Group number	1	2	3	4
*C*_max_ (µg/mL)	1.1 ± 0.4 *^, *a*,*b*^	1.3 ± 0.6 *^, *c*,*d*^	5.4 ± 0.6 *^, *a*,*c*,*e*^	14.9 ± 3.9 *^, *b*,*d*,*e*^
*T*_max_ (min)	5.7 ± 4.4	2.5 ± 1.1	1.7 ± 0.5	5.2 ± 2.6
*AUC*_0–t_ (µg·min/mL)	32 ± 14 *^, *b*^	33 ± 16 *^, *d*^	46 ± 15 *^, *e*^	235 ± 45 *^, *b*,*d*,*e*^

Pharmacokinetic parameters are shown as mean ± standard deviation (*n* = 6). RLA, R-α-lipoic acid; RLA/γ-CD, R-α-lipoic acid/γ-cyclodextrin inclusion complex; *C*_max_, maximum plasma RLA concentration; *T*_max_, time of maximum plasma RLA concentration; *AUC*_0-t_, area under the plasma concentration curve (from initial to last points); po, per os; PL, pylorus ligation; id, intraduodenal. *, Probability (*p*) < 0.05. Statistical analysis was performed among the all groups by using analysis of variance by followed Tukey’s multiple comparison tests. *a*, Group 1 *vs.* 3; *b*, Group 1 *vs.* 4; *c*, Group 2 *vs*. 3; *d*, Group 2 *vs.* 4; *e*, Group 3 *vs.* 4.

### 2.3. Effects of Bile Acid, α-Amylase, and Dissolution on RLA Absorption after Administration as RLA/γ-CD

To clarify the mechanism by which RLA absorption is enhanced by γ-CD inclusion, the following studies were performed and the respective pharmacokinetic parameters were calculated.

**Figure 3:**
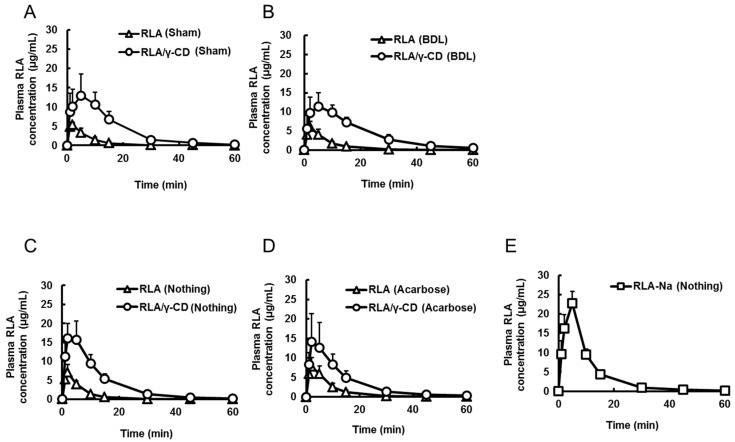
Plasma concentration-time profiles of R-α-lipoic acid after intraduodenal administration of R-α-lipoic acid or R-α-lipoic acid/γ-cyclodextrin inclusion complex; to rats under sham-operation of common bile duct ligation (**A**) or common bile duct ligation (**B**), and to rats co-administered without (**C**) or with acarbose (**D**). The profile after intraduodenal administration of R-α-lipoic acid sodium salt to rats (**E**). Data are shown as mean ± standard deviation (*n* = 6).

Firstly, the effect of bile acid was evaluated (Figure 3A,B). Plasma RLA concentration profiles in the bile duct ligation (BDL) group and the respective sham operation group after intraduodenal administration of RLA/γ-CD were almost identical (Figure 3A,B). The *C*_max_, *T*_max_ and *AUC*_0–__t_ between these groups were not significantly different (Table 3). After intraduodenal administration of non-inclusion RLA, there were no significant differences of the *C*_max_, *T*_max_ or *AUC*_0–__t_ between the BDL group and the sham operation group (Table 3). To confirm the effect of BDL on the biliary excretion of RLA, RLA-Na was intravenously administered (20 mg/kg, 1 mL/kg). The *C*_0_ and *AUC*_0–__t_ were not different between the BDL and sham operation groups (Table 3).

**Table 3:** Pharmacokinetic parameters of R-α-lipoic acid after oral administration or intraduodenal administration of R-α-lipoic acid or R-α-lipoic acid/γ-cyclodextrin inclusion complex to pylorus ligated rats.

Formulation	RLA	RLA/γ-CD	RLA	RLA/γ-CD	RLA-Na	RLA-Na
Route	id	id	id	id	iv	iv
Group number	1	2	3	4	not determined	not determined
Operation	Sham	Sham	BDL	BDL	Sham	BDL
*C*_max_ or *C*_0_ (µg/mL)	5.7 ± 0.8 *^,*a*,*b*^	16.9 ± 5.2 *^,*a*,*c*^	5.8 ± 1.7 *^,*c*,*b*^	11.9 ± 3.5 *^,*b*,*d*^	79.4 ± 20.9	79.2 ± 10.9
*T*_max_ (min)	1.8 ± 0.4 *^,*b*^	5.2 ± 2.9	1.8 ± 0.4 *^,*b*^	6.2 ± 3.2 *^,*b*,*d*^	not determined	not determined
*AUC*_0–t_ (µg·min/mL)	49 ± 16 *^,*a*,*b*^	260 ± 50 *^,*a*,*c*^	54 ± 17 *^,*c*,*d*^	259 ± 55 *^,*b*,*d*^	516 ± 87	540 ± 79

Pharmacokinetic parameters are shown as mean ± standard deviation (*n* = 6). RLA, R-α-lipoic acid; RLA/γ-CD, R-α-lipoic acid/γ-cyclodextrin inclusion complex; RLA-Na, R-α-lipoic acid sodium salt; *C*_max_, maximum plasma RLA concentration; *C*_0_, initial concentration; *T*_max_, time of maximum plasma RLA concentration; *AUC*_0–t_, area under the plasma concentration curve (from initial to last points); id, intraduodenal; iv, intravenous; Sham, sham-operation; BDL, common bile duct ligation. *, Probability (*p*) < 0.05. Statistical analysis was performed among the id groups by using analysis of variance by followed Tukey’s multiple comparison tests. *a*, Group 1 *vs.* 2; *b*, Group 1 *vs.* 4; *c*, Group 2 *vs.* 3; *d*, Group 3 *vs.* 4.

Secondly, the effect of α-amylase in the gastrointestinal tract was evaluated (Figure 3C,D). The activities of α-amylase were 19.3 ± 6.5, 19.2 ± 2.1 and 17.4 ± 6.2 (U/mL) at 10 min after administration of vehicle only, RLA and RLA/γ-CD without acarbose, respectively. On the other hand, the activities were 0.6 ± 0.1and 3.4 ± 0.7 (U/mL) after co-administration of acarbose with RLA and RLA/γ-CD, respectively. Plasma concentration profiles of RLA in the groups given RLA/γ-CD intraduodenally with and without acarbose were almost identical (Figure 3C,D). In addition, the *C*_max_, *T*_max_ and *AUC*_0–__t_ between these groups were not significantly different. Furthermore, after intraduodenal administration of RLA, these parameters were not different between the groups with and without acarbose (Figure 3C,D and Table 4).

Finally, for evaluation of the effects of dissolution, plasma concentration profiles of RLA after intraduodenal administration of RLA/γ-CD and RLA-Na were compared. Excluding the values at 5 min, plasma concentration profiles of RLA/γ-CD and RLA-Na groups were almost identical (Figure 3C,E), and the *C*_max_, *T*_max_ and *AUC*_0–__t_ between these groups were not significantly different (Figure 3C,E and Table 4).

**Table 4:** Pharmacokinetic parameters of R-α-lipoic acid after intraduodenal administration of R-α-lipoic acid, R-α-lipoic acid/γ-cyclodextrin inclusion complex or R-α-lipoic acid sodium salt to rats with or without acarbose.

Formulation	RLA	RLA/γ-CD	RLA	RLA/γ-CD	RLA-Na
Route	id	id	id	id	id
Group number	1	2	3	4	5
Combined-drug	Nothing	Nothing	Acarbose	Acarbose	Nothing
*C*_max_ (µg/mL)	7.0 ± 2.1 *^,*a*,*b*,*c*^	17.1 ± 4.3 *^,*a*,*d*^	8.3 ± 1.9 *^,*d*,*e*^	14.6 ± 7.2 *^,*b*,*f*^	23.8 ± 1.2 *^,*c*,*e*,*f*^
*T*_max_ (min)	1.8 ± 0.4 *^,*c*^	2.8 ± 1.7	3.5 ± 1.6	2.5 ± 1.2	4.5 ± 1.2 *^,*c*^
*AUC*_0-t_ (µg·min/mL)	49 ± 10 *^,*a*,*b*,*c*^	234 ± 47 *^,*a*,*d*^	76 ± 20 *^,*d*,*e*,*g*^	210 ± 57 *^,*b*,*g*^	245 ± 24 *^,*c*,*e*^

Pharmacokinetic parameters are shown as mean ± standard deviation (*n* = 6). RLA, R-α-lipoic acid; RLA/γ-CD, R-α-lipoic acid/γ-cyclodextrin inclusion complex; *C*_max_, maximum plasma RLA concentration; *T*_max_, time of maximum plasma RLA concentration; *AUC*_0–t_, area under the plasma concentration curve (from initial to last points); id, intraduodenal. *, Probability (*p*) < 0.05. Statistical analysis was performed among the all groups by using analysis of variance by followed Tukey’s multiple comparison tests. *a*, Group 1 *vs.* 2, *b*, Group 1 *vs.* 4; *c*, Group 1 *vs.* 5; *d*, Group 2 *vs.* 3; *e*, Group 3 *vs.* 5; *f*, Group 4 *vs.* 5; *g*, Group 3 *vs.* 4.

### 2.4. X-ray Imaging

**Figure 4:**
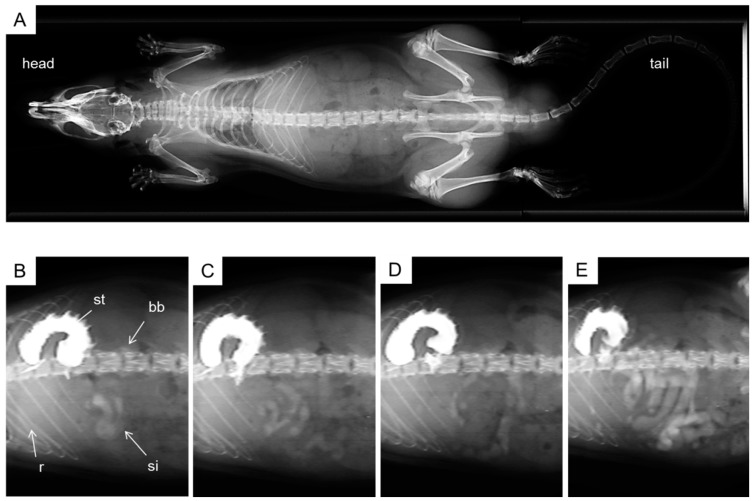
Representative gastrointestinal imaging data after oral administration of contrast medium. Imaging data obtained before administration (**A**); 1 min after administration (**B**); 5 min after administration (**C**); 15 min after administration (**D**) and 30 min after administration (**E**). bb, backbone; r, rib; st, stomach; si, small intestine.

Typical images after oral administration of Gastrografin^®^ to rats (2 mL/kg, *n* = 3) are shown in Figure 4. No opacity in the pre-dose image was observed. On the other hand, 1 min after administration the stomach was strongly opaque from the contrast medium, while the small intestine was slightly opaque. Over an interval of 30 min, the opacity was gradually delivered into the small intestine. The viscosity of the contrast medium (9.03 mPa·s) was similar to that of 0.5% (*w*/*v*) CMC-Na solution (8.28 ± 0.05 mPa·s).

## 3. Discussion

Recently, Ikuta *et al*., reported that RLA/γ-CD was suitable for pharmaceutical formulation in consideration of pharmaceutical processing [24]. On the other hand, there have been many reports that CD inclusion can achieve good oral bioavailability of poorly absorbable drugs [27]. Specifically, several studies have revealed that γ-CD inclusion complexes enhanced the bioavailability of drugs such as digoxin in dogs, diazepam in rabbits, and artemisinin and coenzyme Q10 in humans [28,29,30,31], but there have been no studies to test RLA. In our present study, comparing the AUC0-120 of RLA after oral administration of α-, β- and γ-CD inclusion complexes to rats, it was found that RLA/γ-CD showed the highest plasma exposure among the groups (Figure 1 and Table 1). This result demonstrated that RLA by γ-CD inclusion was the preferable complex to the other CDs in the light of oral absorption. Then, we examined in detail the mechanisms by which γ-CD inclusion could enhance absorption of RLA.

To examine the absorption from the stomach, non-inclusion RLA or RLA/γ-CD was administered to rats after pylorus ligation; plasma concentrations in excess of the endogenous level of RLA were detected only 2 min after dosing with either formulation (Figure 2A) [32]. This result indicates that RLA is rapidly absorbed from the stomach regardless of the formulation in rats. Furthermore, the concentration profiles of the two formulations were almost equal in extent and velocity. A similar observation was reported in an *in situ* absorption study by Peter and Borbe [33]. Moreover, the gastric pH of humans in the fasted state is well known to be approximately 1.2, whereas that of rats was reported to be approximately 4.0 [34,35]. Therefore, RLA in the rat stomach was stable enough, so that there was no difference in the absorption from the two formulations. When administered to humans, RLA/γ-CD might be more stable in the stomach than RLA itself, and could achieve higher exposure.

On the other hand, the absorption of RLA was greater after intraduodenal administration of RLA/γ-CD than after administration of non-inclusion RLA (*p* < 0.05). Furthermore, from the result of the X-ray imaging using a contrast medium (Figure 4), it was suggested that both formulations reached the small intestine within a few minutes after oral administration. These results showed that the difference in absorption between RLA/γ-CD and non-inclusion RLA was occurred mainly in small intestine, even at less than 5 min after oral administration (Figure 4B,C). Of course, however, a large portion of the administered dose was retained in the stomach at 30 min, so that the *AUC*_0–t_ and *C*_max_ after oral administration was lower than those values after intraduodenal administration. These results indicate that some factors in the small intestine enhance RLA absorption by γ-CD inclusion complex.

In 2012, Uekaji *et al*. reported that coenzyme Q10, as a guest molecule in γ-CD complexes, was replaced with bile acids, was liberated and formed micelles also with bile acids as a mechanism for enhancement of absorption [36]. If the same process also occurs in the rat intestine in the case of RLA, liberation of RLA molecules could be increased and absorbed effectively. However, the AUC of RLA after intraduodenal administration of RLA/γ-CD was not different between the BDL group and the respective sham operation group, indicating that bile acid wasn’t associated with the absorption of RLA after liberation from γ-CD.

The inclusion of a guest molecule in, and its liberation from, CD was reversible and held in equilibrium [37]. Therefore, a γ-CD, which releases a guest molecule, would be present at definite proportions in the small intestine. If γ-CD in the small intestine were digested to maltose or glucose by pancreatic amylase, liberation of RLA molecules from γ-CD probably would be promoted, and plasma RLA concentration could be increased. Therefore, we elucidated whether α-amylase activity had an effect on the liberation of RLA from γ-CD and on its absorption. However, the *AUC*_0-t_ of RLA after intraduodenal administration of RLA/γ-CD was not different between groups treated with and without acarbose, an α-amylase inhibitor. This result indicated that α-amylase activity wasn’t associated with the absorption of RLA after its liberation from γ-CD, either.

The pharmacokinetic parameters after intraduodenal administration of RLA/γCD and water-soluble RLA-Na were almost comparable. That is to say, RLA/γ-CD could be dissolved immediately in small intestinal fluid, and RLA might be liberated continuously from the complex. From Trentin’s study on the stability constant of lipoate anion with CDs [38], γ-CD had a lower stability constant than α- or β-CD, because γ-CD has the biggest cavity among them. According to these results, we regarded the dissolution process to be a key factor in the mechanism for enhancement of RLA/γ-CD, and not the liberation of RLA from γ-CD.

Furthermore, we also considered whether CD increases the paracellular permeability of intestinal membranes by opening the tight junctions. Several reports have revealed that the permeability of nasal or cutaneous membranes to drugs was enhanced by β-CD [39,40]. Those studies, however, required the pre-treatment for a few hours to open the tight junctions. However, RLA was rapidly absorbed after drug administration, and thereby this mechanism was thought to be not associated with the enhancement of RLA absorption. In addition, intestinal metabolism before the absorption was also considered. Thus, the protective effect of CDs from metabolism by bacteria or enzymes should be clarified by *in vitro* experiments.

## 4. Experimental Section

### 4.1. Chemicals and Reagents

RLA (purity > 99.0%) and RLA-Na (purity > 99.5%) were purchased from Changshu Fushilai Medicine and Chemical Co., Ltd. (Changshu, China). R/S-LA (purity > 98.0%) was purchased from Sigma-Aldrich Production GmbH (Buchs, Switzerland). RLA/α-CD (1 mg RLA/6.71 mg complex, containing 14.9% RLA), RLA/β-CD (1 mg RLA/7.81 mg complex, containing 12.8% RLA), and RLA/γ-CD (1 mg RLA/8.77 mg complex, containing 11.4% RLA) were prepared at CycloChem Bio Co., Ltd. (Kobe, Japan) [24]. Acarbose (purity > 95.0%) was purchased from Wako Pure Chemical Industries, Ltd. (Osaka, Japan). Gastrografin^®^ was purchased from Bayer (Leverkusen, Germany). All other chemicals and reagents were commercially available and of analytical grade or higher.

### 4.2. Animals

Male Sprague-Dawley (SD) rats were obtained from Japan SLC Inc. (Hamamatsu, Japan), and used at the age of 8 weeks (230–270 g) after at least 1 week of acclimatization. All rats were housed in a temperature- (23 ± 1 °C) and humidity- (55% ± 5%) controlled room with 12 h light/dark cycle. Water and food (Labo MR stock, Nosan Corporation, Yokohama, Japan) were available ad libitum throughout the study except as described below. The rats were fasted for at least 12 h before drug administration and drugs were administered under isoflurane anesthesia regardless of the administration route. After the experiments, the rats were killed by exsanguination also under anesthesia. All rats were handled in accordance with the institutional and national guidelines for the care and use of laboratory animals.

### 4.3. Drug Administration

Oral and intravenous administration: Non-inclusion RLA or RLA/CDs suspended in 0.5% (*w*/*v*) carboxymethylcellulose sodium salt (CMC-Na) solution were orally administered to rats (20 mg RLA/kg, 2 mL/kg, *n* = 6) by using a feeding needle. RLA-Na dissolved in saline was intravenously administered to rats (5 mg or 20 mg RLA/kg, 1 mL/kg, *n* = 6) via the caudal vein.

Oral administration under pylorus ligation: The pylorus of each rat was ligated by the technique of Blair *et al*. with minor modification as follows [41]. Under isoflurane anesthesia, the abdomen was opened, the pylorus was slightly lifted and ligated with cotton thread, and then the incision was closed immediately with suture and an adhesive was applied. After surgery, non-inclusion RLA or RLA/γ-CD was orally administered to the rats (20 mg RLA/kg, 2 mL/kg, *n* = 6). The absorption from the stomach was evaluated based on the results of this experiment.

Intraduodenal administration: Just before pylorus ligation in the course of the operation mentioned above, non-inclusion RLA, RLA/γ-CD or RLA-Na was injected from a syringe with a 21-gauge needle into the duodenum of the rats through the gastric corpus (20 mg RLA/kg, 2 mL/kg, *n* = 6). Immediately after the injection, the pylorus was tightly ligated with suture to prevent reflux of the compound back into the stomach. The incision was closed with suture and adhesive was applied. In the experiment to evaluate the α-amylase effect, acarbose (10 mg/kg) dissolved in 0.5% (*w*/*v*) CMC-Na solution was co-administered with non-inclusion RLA or RLA/γ-CD (20 mg RLA/kg, 2 mL/kg, *n* = 6).

### 4.4. Bile Duct Ligation

The bile duct ligation (BDL) was performed by the technique of Cameron *et al*. with minor modification as follows [42]. Under isoflurane anesthesia, the abdomin was opened in the same way as mentioned above. The duodenum was pulled to expose the common bile duct, which was then doubly ligated with thread. The incision was closed with suture and adhesive was applied. The same procedure but without the ligation was performed as a sham operation. Rats were used 48 h after the operation to deplete the bile salts from the small intestine [43].

### 4.5. Assay of α-Amylase Activity in the Intestinal Lumen

Preparation of the samples for the assay was performed by the technique of Jin *et al*. with minor modification [44]. Briefly, 10 min after drug administration, the intestinal contents were collected from a 10-cm segment of the tract from the duodenum to the jejunum, and were diluted with 10-fold its volume of PBS (pH 7.0). The diluted samples were homogenized with a glass Teflon homogenizer for 1 min and sonicated for 1 min with three cycles at 30 s intervals. The homogenates were centrifuged at 10,800× *g* and 4 °C for 20 min. The activity of α-amylase in the supernatant was measured by α-Amylase Assay kit (Kikkoman Biochemifa Company, Tokyo, Japan).

### 4.6. Blood Collection

Blood was withdrawn from the external jugular vein using heparinized syringes under isoflurane anesthesia at 0 (predose), 1, 2, 5, 10, 15, 30, 45, 60, 90 and 120 min or at the same time points until 60 min after drug administration. The collected blood was centrifuged at 3000× *g* and 4 °C for 10 min to obtain plasma. Plasma was stored at −20 °C until the analysis.

### 4.7. Determination of Plasma RLA Concentration by LC-MS/MS

The LC-MS/MS system consisted of API 3200™ (AB SCIEX, Framingham, MA, USA) interfaced with a Shimadzu Prominence HPLC system (Shimadzu, Kyoto, Japan). The HPLC system consisted of a LC-20AD binary pump, DGU-20A3 degasser, SIL-20A autosampler, CTO-20A column oven, CBM-20A system controller. The measurement was performed using the method of Chen *et al*. with minor modification [45]. Briefly, 90 µL of acetonitrile containing 0.1% (*v*/*v*) formic acid and 100 ng/mL of furosemide as an internal standard was added to a 10 µL plasma sample. After mixing, the sample was centrifuged at 10,800× *g* and 4 °C for 10 min. Two µL of the supernatant was applied onto the LC-MS/MS system. The HPLC was fitted with a CAPCELL PAK C18 AQ column (3 µm, 2.0 × 50 mm, Shiseido, Tokyo, Japan), and chromatography was performed using a gradient elution program at a flow rate of 0.3 mL/min. The column temperature was maintained at 40 °C. The mobile phase consisted of 0.1% (*v*/*v*) formic acid/water (A) and 0.1% (*v*/*v*) formic acid/acetonitrile (B). The gradient program was as follows: hold the ratio of A/B at 70/30 for 0.1 min, increase B linearly from 30% to 95% between *t* = 0.1 and 0.2 min, decrease B linearly from 95% to 30% between *t* = 0.2 and 2.0 min, then hold the ratio of A/B at 70/30 until *t* = 5.0 min. The analyte and internal standard from the column were detected by the negative ion mode, and analyzed by multiple reaction monitoring mode of the transitions *m*/*z* 207.0 to 173.2 for RLA and *m*/*z* 329.1 to 204.6 for furosemide.

### 4.8. X-ray Imaging

Digital imaging of the upper gastrointestinal tract was performed after oral administration of a contrast medium as follows. The X-ray imaging was performed using a Latheta LCT-200 (Hitachi-Aloka, Tokyo, Japan) in general radiography mode. The tube voltage was set at 50 kV, the tube current was constant at 0.5 mA, and the axial field of view was selected as 80 mm. Under isoflurane anesthesia, an ionic contrast medium, Gastrografin^®^ was orally administered to the rats (2 mL/kg, *n* = 3) by using a feeding needle. At 0 (predose), 1, 2, 5, 10, 15 and 30 min after administration, the imaging data were obtained.

### 4.9. Measurement of Viscosity

The viscosity of 0.5% (*v*/*v*) CMC-Na solution and of Gastrografin^®^ were measured with a DV2T Viscometer (Brookfield Engineering Laboratories, Middleboro, MA, USA) at 37 °C.

### 4.10. Pharmacokinetics Analysis

The maximum plasma concentration (*C*_max_) and time to peak (*T*_max_) were obtained directly from the individual plasma concentration-time profiles. Area under the plasma concentration *vs.* time curve (AUC) of RLA was estimated using the trapezoidal rule. To estimate the exposure to RLA, ratio of *AUC*_po_/*AUC*_iv_ from *t* = 0 min to *t* = 120 min was calculated from the following equation.

$${\text{AUC}}_{\text{po}}/{\text{AUC}}_{\text{iv}}\left(\text{\%}\right)\text{= }\left({\text{AUC}}_{\text{0–t, po}}{\text{ × Dose}}_{\text{iv}}\right)/\left({\text{AUC}}_{\text{0–t, iv}}{\text{ × Dose}}_{\text{po}}\right)\text{×100}$$

### 4.11. Statistical Analysis

Excluding the values of *AUC*_po_/*AUC*_iv_ and Gastrografin^®^ viscosity, parameters are presented as arithmetic mean ± standard deviation. The pharmacokinetic parameters were compared with one-way analysis of variance followed by Tukey’s multiple comparison tests. Differences were considered statistically significant when *p* < 0.05.

## 5. Conclusions

We revealed that the γ-CD inclusion complex was more suitable for oral administration of RLA than those with the other natural CDs. When administered as RLA/γ-CD inclusion complex, RLA can be absorbed from the small intestine effectively by easily dissolving in the lumen of the intestine. These results also suggest that the absorption of RLA in humans could be increased by taking a RLA/γ-CD formulation. Hence, RLA/γ-CD is concluded to be an appropriate formulation for supplying RLA as a drug or nutritional supplement in the light, not only of formulation stability, but also of absorption performance. However, although there were few differences in structure among the three CDs, α-CD and β-CD did not enhance RLA absorption. More detailed studies about RLA/α-CD and RLA/β-CD or about CD inclusion complex with other guest molecules will be needed to clarify the property of CDs as a drug delivery material.
